# Outcomes of Brood Parasite–Host Interactions Mediated by Egg Matching: Common Cuckoos *Cuculus canorus* versus *Fringilla* Finches

**DOI:** 10.1371/journal.pone.0019288

**Published:** 2011-04-29

**Authors:** Johan Reinert Vikan, Frode Fossøy, Esa Huhta, Arne Moksnes, Eivin Røskaft, Bård Gunnar Stokke

**Affiliations:** 1 Department of Biology, Norwegian University of Science and Technology (NTNU), Trondheim, Norway; 2 Rovaniemi Research Station, Finnish Forest Research Institute, Rovaniemi, Finland; Smithsonian Institution National Zoological Park, United States of America

## Abstract

**Background:**

Antagonistic species often interact via matching of phenotypes, and interactions between brood parasitic common cuckoos (*Cuculus canorus*) and their hosts constitute classic examples. The outcome of a parasitic event is often determined by the match between host and cuckoo eggs, giving rise to potentially strong associations between fitness and egg phenotype. Yet, empirical efforts aiming to document and understand the resulting evolutionary outcomes are in short supply.

**Methods/Principal Findings:**

We used avian color space models to analyze patterns of egg color variation within and between the cuckoo and two closely related hosts, the nomadic brambling (*Fringilla montifringilla*) and the site fidelic chaffinch (*F. coelebs*). We found that there is pronounced opportunity for disruptive selection on brambling egg coloration. The corresponding cuckoo host race has evolved egg colors that maximize fitness in both sympatric and allopatric brambling populations. By contrast, the chaffinch has a more bimodal egg color distribution consistent with the evolutionary direction predicted for the brambling. Whereas the brambling and its cuckoo host race show little geographical variation in their egg color distributions, the chaffinch's distribution becomes increasingly dissimilar to the brambling's distribution towards the core area of the brambling cuckoo host race.

**Conclusion:**

High rates of brambling gene flow is likely to cool down coevolutionary hot spots by cancelling out the selection imposed by a patchily distributed cuckoo host race, thereby promoting a matching equilibrium. By contrast, the site fidelic chaffinch is more likely to respond to selection from adapting cuckoos, resulting in a markedly more bimodal egg color distribution. The geographic variation in the chaffinch's egg color distribution could reflect a historical gradient in parasitism pressure. Finally, marked cuckoo egg polymorphisms are unlikely to evolve in these systems unless the hosts evolve even more exquisite egg recognition capabilities than currently possessed.

## Introduction

Numerous antagonistic species interact via matching of phenotypes [1], [2], [3], [4], [5]. Some of the prime examples of this kind are found among avian brood parasites, such as the common cuckoo (*Cuculus canorus*) (hereafter cuckoo), and their hosts. In these interactions, the outcome of a parasitic event is often determined by the match between cuckoo and host eggs [6]. Since this situation can give rise to strong associations between fitness and egg phenotype in both species, and because the heritabilities involved are high [7], [8], [9], much of the coevolutionary dynamics of these interactions is likely to involve egg phenotypes. Indeed, comparative evidence implicates coevolution as the main driver behind the high egg phenotype diversity found in some hosts of specialized brood parasites [10], [11]. However, our present insight in the outcomes of egg phenotype coevolution rests almost exclusively on mathematical models and theoretical arguments [12], [13], [14], [15], [16], [17], [18], [19]. Theoretical predictions cover a wide range of scenarios, from matching equilibria (equal mean phenotypes) to non-matching equilibria, coevolutionary cycles and stable point polymorphisms, depending on the specific assumptions made about the structure of genetic variance, the levels of inheritance, and the presence of stabilizing selection. This diversity of theoretical outcomes warrants detailed empirical investigations of egg phenotype distributions in different host parasite-systems [18]. Once attained, such data can be used to assess the opportunity for reciprocal selection, determine the mode of selection acting on host and parasite, and evaluate parasite and host phenotypes in relation to predicted optima. Such approaches are important because they will facilitate more informed discussions about evolutionary directions.

**Figure 1:**
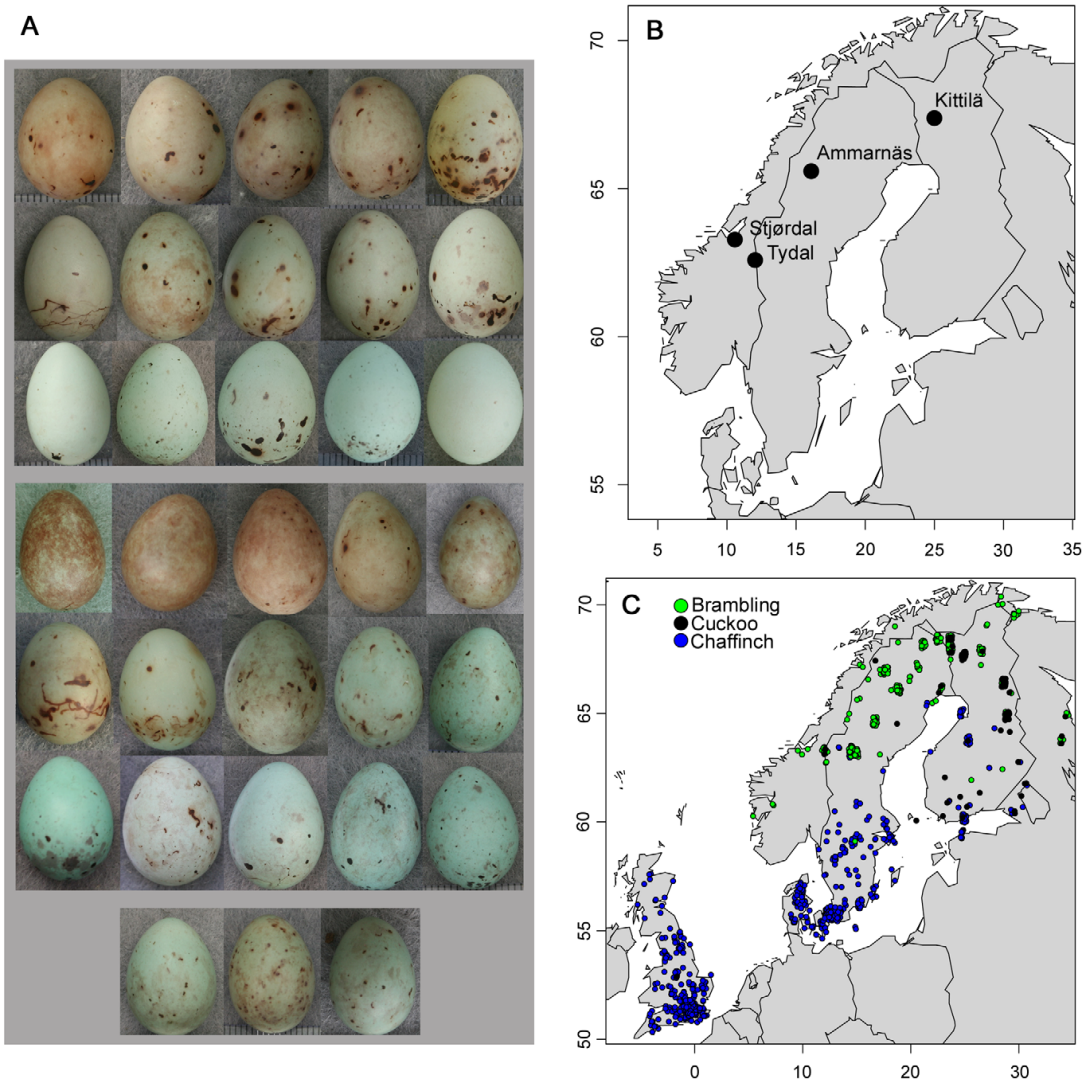
Study populations, phenotypic variation of brambling and chaffinch eggs, and geographical distribution of museum clutches. A: range of egg colors of chaffinch (rows 1-3) and brambling clutches (rows 4-6). Each egg comes from a different clutch. The bottom row gives examples of cuckoo eggs found in brambling nests in Kittilä. B: location of study populations. C: geographic origin of museum clutches. The points are slightly jittered for plotting purpose. The maps were drawn using the *maps* package for R [88].

In this study, we compare patterns of variation in egg color distributions within and between two closely related cuckoo hosts, the brambling (*Fringilla montifringilla*) and the chaffinch (*F. coelebs*). The cuckoo is known to comprise specialized female lineages (called gentes) which in many cases have evolved eggs that tend to mimic those of their respective hosts, resulting in an astonishing diversity of egg types [20], [21], [22], [23]. The two *Fringilla* finches show the same wide range of egg colors, including pure blue, green, reddish-grey and dark olive-brown clutches (Figure 1A), and are thus apt for investigating fitness in relation to variation in egg phenotypes. Since egg rejection probability is strongly influenced by the match between host and cuckoo egg [24], [25] and acceptance of the cuckoo egg leads to a massive reduction in nestling production [6], both finches show sufficient variation between clutches to generate significant fitness differences among individuals in parasitized populations. Moreover, both species possess all the basic ecological features that characterize prime cuckoo hosts. In particular, both build arboreal and shallow nest cups and often make up 20-50% of their respective breeding passerine communities, and raise their chicks on a protein diet suitable for cuckoos [26], [27], [28], [29], [30]. In addition, both show highly developed egg recognition abilities throughout at all sites where they have been tested and both respond aggressively towards adult cuckoos [25], [31], [32], [33], [34]. Finally, the existence of a cuckoo egg morph resembling *Fringilla* eggs has been well documented [20], [22], [25], [35], [36]. Parsimony therefore suggests that the two finches have a history as important hosts for the cuckoo in Europe [34], [37]. At the same time, their natural histories imply that they differ markedly in their ability to evolve in response to adapting cuckoos, which leads to different predictions regarding coevolutionary outcomes [12], [13], [17], [38], [39], [40].

The brambling is the northern boreal ecological counterpart of the chaffinch [28]. It employs an opportunistic breeding strategy for exploitation of favourable but unpredictable breeding opportunities [29], [41], and therefore has a very low breeding site fidelity [42], [43], [44] which is indicative of high amounts of gene flow possibly swamping local selection [45]. Out of 1945 breeding adults marked in Finnish and Swedish Lapland, only 11 individuals (0.6%) have subsequently been recaptured on the same breeding site, whereas none out of 2300 marked juveniles have been recaptured [42,43, Å. Lindström, unpublished data]. Moreover, recoveries at different breeding sites indicate that adult bramblings may breed at sites up to 600 km apart in different years [43]. By contrast, the chaffinch exhibits marked breeding site fidelity [42], [44], [46], possibly reflecting the more predictable ecological conditions of a southern distributional range [47]. In Finnish Lapland, 39.3% of marked breeding chaffinches were found to nest again at the same site the following year [42]. The potential evolutionary consequences of these differences are manifested in the subspecies-structure of the two species. While the chaffinch has a marked geographical variation comprising several subspecies (7 in the *coelebs* group), no subspecies has so far been recognized in the brambling [27], [28].

Thus, theory prescribes that the extent of egg phenotype evolution should differ significantly between the two species because, all else being equal, the ability to evolve in response to adapting cuckoos should be markedly stronger in the chaffinch. Interactions that are mediated by phenotype-matching are likely to involve evolution of increasingly bimodal host phenotype distributions as a response to disruptive selection imposed by mimetic parasite phenotypes [17]. We should therefore (1) expect to find stronger bimodality in the egg colors of the chaffinch than in the brambling. The first aim of this study is to investigate the possible occurrence of such differences. In order to achieve this, we use a tetrahedral color space model to compare the egg color distributions of the two species, evaluate the current opportunity for disruptive selection on brambling egg colors, and visualize the direction of predicted evolutionary change in color space.

Secondly, we evaluate cuckoo eggs in relation to predicted phenotypic optima for three distant brambling populations in Fennoscandia (one currently parasitized and two non-parasitized). Given the nomadic nature of bramblings, we predict (2) that cuckoos should be equally well adapted to the egg phenotype distributions of the brambling in the three areas. In other words, we expect little geographical variation in the eggs of cuckoos specializing on bramblings. In general, the geographical scale at which we find noticeable variation in brambling and cuckoo egg phenotypes should be very large in this system.

Finally, (3) we examine if the range of the coevolutionary trajectory is likely to extend beyond the stage of increasing host variance. One step in this direction is to evaluate whether evolution on part of the host (bimodal egg color distribution) can give rise to corresponding adaptive peaks for cuckoo egg appearance [16], [48]. To this end, we estimate average rejection probabilities for a wide range of potential cuckoo egg phenotypes, and use these probabilities to sketch the forms of selection imposed by the hosts' egg color distributions and relevant subsets of these distributions.

## Methods

### Ethics statement

Our research followed guidelines of the Animal Behaviour Society for ethical use of animals in research, and permissions for the protocols involved in the fieldwork were provided by Lapland Regional Environment Centre (permit numbers LAP-2005-L-41-254, LAP-2005-L-666-254/1.3.2006, LAP-2008-L-191-254), The Finnish Forestry agency (permit numbers 1737/42/2005, 1207/41/2006, 2296/662/2008), The Swedish Committee for Animal Research (permit number A 35-08), and the Norwegian Directorate for Nature Management (permit numbers 2008/1524 Art-VI-ID,05/2580 ART-VI-ARES, 2007/1177 ART-VI-JAA).

### Field data

The brambling was studied in three areas in Fennoscandia (Figure 1B): Tydal in Central Norway (63°N, 12°E, 2006-2008), Ammarnäs in Northern Sweden (66°N, 16°E, 2008), and Kittilä in Northern Finland (68°N, 25°E, 2005–2008). The Kittilä population is the only one currently parasitized. Data on host egg coloration were obtained from 47 clutches in Tydal, 97 clutches in Ammarnäs, and 88 clutches in Kittilä, whereas data on cuckoo egg coloration were obtained from five cuckoo eggs measured in Kittilä. Genetic analyses on four of them and volume and shape analyses show that these eggs stem from at least four different females (own unpublished data). The chaffinch was studied in an unparasitized population in Stjørdal, Central Norway (63°N, 11°E, 2007–2008), where data from 157 clutches were obtained.

### Museum data

In addition to field data, we also collected data on chaffinch, brambling, and cuckoo egg appearance from clutches held in the collections of British Museum (Natural History), Tring, Great Britain; Museum of Natural History, Gothenburg, Sweden; Museum of Natural History, Copenhagen, Denmark; Finnish Museum of Natural History, Helsinki, Finland, and Swedish Museum of Natural History, Stockholm, Sweden. Data on 343 brambling and 625 chaffinch clutches collected during the period 1839–1991 were included in the analyses. Figure 1C shows the geographical distribution of the clutches.

Based on inspection, cuckoo eggs were classified to belong to a *Fringilla* morph [22] if the egg was clearly within the range of variation of brambling and chaffinch eggs (N = 72). These cuckoo eggs were laid in clutches of brambling (N = 47), chaffinch (N = 10), willow warbler (*Phylloscopus trochilus*) (N = 5), robin (*Erithacus rubecula*) (N = 2), spotted flycatcher (*Muscicapa striata*) (N = 2), reed bunting (*Emberiza schoeniclus*) (N = 1), tree pipit (*Anthus trivialis*) (N = 1), rustic bunting (*Emberiza rustica*) (N = 1), chiffchaff (*Phylloscopus collybita*) (N = 1), yellow wagtail (*Motacilla flava*) (N = 1), and one unknown host species. In all of the non-*Fringilla* hosts, the sampling locality was within Northern Fennoscandia and indicated sympatry with either chaffinch or brambling.

Cuckoo eggs found in the clutches of chaffinches or bramblings that obviously belonged to a different cuckoo egg morph (such as *Anthus* or *Motacilla* [22]) were not included in the analyses. There are two main reasons for this delimitation. Firstly, in the context of egg phenotype coevolution, cuckoo eggs that are outside the trait space of the host are likely to contribute little to selection on host egg appearance, because such eggs are nearly always rejected by both hosts, irrespective of the host's own egg type. We have conducted a total of 66 egg rejection experiments where the parasitic egg came from a non-*Fringilla* species, and 92% of those eggs were rejected (19/19 experiments with bramblings and 42/47 experiments with chaffinches). Secondly, the occurrence of such cuckoo eggs may differ between the two *Fringilla* hosts for reasons that are completely unrelated to a coevolutionary process, for example because of differences in host community composition (host-specific gentes may accidentally lay eggs in other hosts' nests [22]).

Data on sampling location was available for 581 chaffinch clutches, 341 brambling clutches, and 68 cuckoo eggs. In 771 cases (438 chaffinch, 270 brambling, and 63 cuckoo), the clutch label contained information about city/municipality or a specific site within a city/municipality. In the remaining 218 cases (5 cuckoo, 143 chaffinch, 70 brambling), the label contained information about the county/province/shire in which the clutch was collected. In these cases, the site of collection was defined as the centre of the county/province/shire (chosen by visual inspection of the map). Data on the year of collection was available for 600 chaffinch clutches, 333 brambling clutches, and 72 cuckoo eggs.

### Egg Experiments

We carried out egg exchange experiments across all study populations in order to obtain the host discrimination function which best describes the relationship between the color contrast between host and parasitic eggs and the probability of egg rejection. In this study, the host discrimination function is used to estimate average survival probabilities for host and cuckoo eggs. As experimental parasitic eggs, we used real brambling and chaffinch eggs. Hence, the host discrimination function obtained applies to differences that occur within the boundaries of the trait space of the two hosts. A total of 288 successful experiments were recorded (137 with bramblings (14 of the parasitic eggs where chaffinch eggs) and 151 with chaffinches (all parasitic eggs were chaffinch eggs)). The result of each experiment was classified as either rejection (parasitic egg ejected) or acceptance (parasitic egg incubated for at least five days). We have shown in two separate studies that previous experimental parasitism does not affect the probability of rejection of a parasitic egg added later in the same breeding attempt [25], [49]. We therefore included seventy-four experiments that were replicates at the individual level. In all cases where two experiments were carried out on the same individual, two different parasitic eggs were used (one of high contrast and one of low-medium contrast). For further details about the egg experiments see [24], [25], [49].

### Measurements of Egg Shell Reflectance

We used a spectrophotometer to obtain reflectance spectra of the ground color of the eggs. The measurement procedures are detailed elsewhere [24], [25], [49]. Eggs collected in the field were all fresh when measured. One random egg was measured in each clutch, which is justified by the extraordinary low intraclutch variation found in these two species [34], [50]. Four (n = 1080) or eight (n = 126) measurements were taken from each egg, which is sufficient to describe background coloration adequately in these species [49]. We therefore calculated an average spectrum from the four (eight) measurements, and used these spectra for the subsequent analyses.

### Color Space, Egg Color Contrasts, and Egg Color Distributions

In order to analyze egg colors and egg color distributions we applied a tetrahedral color space model which has been strongly advocated for use in studies of color evolution in tetrachromats [51]. All formulas used for the color space calculations are detailed in [51], [52]. The main advantages of the this color space model are that it makes very few assumptions, it is pragmatic and quantitatively precise, and it provides a transportable scale of measurement that can be compared among independent analyses (unlike the principal component analyses which hitherto has dominated egg color research) [51]. The position of any color in the color space is determined by the relative stimulation of the four retinal cone types by the reflectance spectrum under idealized light conditions. Thus, the position of each egg color is given by a set of relative cone stimulation values {uv, s, m, l}. The four vertices of the tetrahedron correspond to exclusive stimulation of the ultraviolet-sensitive (uv), short-wavelength-sensitive (s), medium-wavelength-sensitive (m), and long-wavelength-sensitive cone photoreceptors (l) (see Figure 1 in [51]). For these calculations, we used the average of spectral sensitivity curves for UVS – type retinas from Endler and Mielke [53, available in their supplementary online material]. Following [51], the cone stimulation values were normalized to sum to 1 and then transformed into Cartesian coordinates {x, y, z}. The chosen transformation places the achromatic point of equal cone stimulation at the origin and the uv-vertex along the z-axis (see Figure 1 in [51]).

The color contrast (ΔT_C_), i.e. level of mimicry, between any two eggs was calculated as the Euclidean distance between the two egg colors in tetrahedral color space. Hue and saturation, which are fundamental color aspects, were obtained by deriving the spherical coordinates θ, φ, and r of the color vector {x, y, z} (see Figure 1 in [51]). The hue of a color is defined as the direction of the color vector, and is therefore given by the angular displacement of the color vector from the positive x-axis (θ 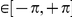), which runs between the m (green) and l (red) vertices of the tetrahedron, and the angular displacement from the horizontal xy-plane (Φ 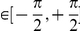), which equals the uv-component of hue [51]. θ and Φ are analogous to longitude and latitude, respectively. A convenient and heuristic way to visualize the distribution of hues for a sample of clutches is to map the hues onto a unit sphere centered at the achromatic origin and derive their two-dimensional Robinson projections (*sensu* [54]).

Saturation describes how different a color is from achromatic white/black. The length of the color vector is therefore a measure of saturation. This means that colors that differ in saturation but not in hue are positive scalar multiples of the same color vector.

Brightness is not part of the tetrachromatic color space, but contrast in brightness can be an important recognition cue for the host in some circumstances [55], [56]. However, this does not appear to be the case with bramblings and chaffinches [24,25, see Results section], and we therefore only focus on color (i.e. variation between reflectance spectra independent of intensity) in this study.

### Estimating the Form of Selection on Cuckoo and Host Egg Color

In order to evaluate the direction of selection on brambling egg phenotypes we calculated the average color contrast for each clutch based on pairings with real cuckoo eggs. For brambling clutches measured in the field we used the five cuckoo eggs found in brambling nests in Kittilä. For museum clutches we used 72 *Fringilla* type cuckoo eggs (see above). In order to evaluate how close cuckoo eggs are to the optimal phenotype for a population, we calculated pair wise color contrasts between all clutches in a population, and the best achievable mimetic egg (i.e. optimal cuckoo egg type) for that population was taken to equal the clutch with the lowest average color contrast. The average rejection probability of optimal cuckoo egg types was estimated using the host discrimination function.

In order to evaluate the potential forms of selection imposed on cuckoo eggs by the hosts, we sampled 3000 potential cuckoo egg colors from within the tetrachromatic color space of each host that were evenly distributed for hue and saturation and then calculated the average acceptance probability of each of these eggs. In order to assess the effects of increasing host bimodality on the adaptive landscape of the cuckoo, we also simulated that the potential cuckoo eggs were facing appropriate subsets of the host's current distribution (i.e. increasingly bimodal host distributions).

### Statistical Analyses

All calculations and analyses were carried out in R2.8.1. We used a binomial logistic regression (logit link) to obtain parameter estimates for prediction of egg rejection probability. We used distance based permutation tests for comparisons of egg color distributions within and among species, and for evaluating changes in egg color distributions along latitudinal/longitudinal gradients. Mantel tests [57] were used to examine the degree of concordance between geographical distances and color distances (ΔT_C_). Mantel tests were performed using the vegan package [58]. Year of collection was controlled for in partial Mantel tests. There was a weak temporal trend in the egg colors of museum eggs (explaining 1.2% of the total variation), and the effect was similar in all three species (own unpublished results). Furthermore, there was no relationship between year of collection and geographical coordinates of sampling locality (own unpublished results). Finally, the direction of the temporal trend was in close agreement with what one should expect regarding the effects of storage and ageing (JR Vikan, unpublished results). However, since the aim of this study is not to determine what factors contribute to the temporal effect, the crucial point to emphasize is that museum eggs can be used to conduct meaningful comparisons of geographical variation in the three species.

Geographical distances between localities were estimated using the *rdist.earth* function in the fields package [59], which returns Great circle distances from longitude/latitude data. We used permutational MANOVAs (function *adonis* in vegan package) to examine effects of latitude/longitude on color space location. This procedure partitions the variation inherent in distance matrixes (in our case matrixes in which elements are color distances between eggs), and uses permutation tests to inspect the significances of those partitions (so called pseudo-F tests). The *adonis* function also returns a partial-R^2^, which is an estimate of the proportion of variance explained by the variable.

We used a multivariate analogue of Levene's test to test for differences in egg color variances across species and populations (Multivariate homogeneity of group dispersions, MHGD). The procedure works by first calculating the distances between egg colors and respective group centroids (function *betadisper* in vegan package). The magnitudes of these distances are then compared between groups using ordinary ANOVA.

Finally, we used the Euclidean distance version of a multiresponse permutation procedure (MRPP [60]) to test for overall differences in the egg color distributions of chaffinches and bramblings. MRPP is closely related to permutational MANOVA, since both are permutation tests based on distances among multivariate observations [61]. In the MRPP procedure, the effect size of the difference between distributions is given by a disparity statistic (K), which takes into account differences in group centroids, variances, skewness, kurtosis and shape [53].

Number of permutations was set to 1000 for the Mantel and partial Mantel tests, and 10000 for the MRPP and permutational MANOVAs.

## Results

### The Host Discrimination Function

**Figure 2:**
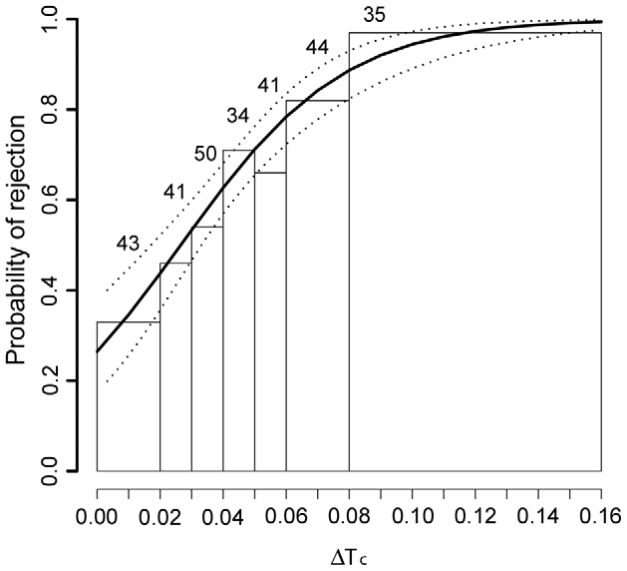
Host discrimination function. Bar plot showing the relationship between rejection rate and color contrast (ΔT_C_) between host and parasitic eggs. Superimposed is the host discrimination function obtained from a univariate logistic regression on the probability of rejection (95% confidence interval indicated by dotted lines). For plotting purposes the width of the bars for some groups were adjusted to obtain similar sample sizes across groups. Sample sizes for each group depicted above the bars.

The egg exchange experiments revealed no significant interactions between species and the four different measures of contrast between eggs (ΔT_C_, brightness contrast, volume contrast, shape contrast). Furthermore, ΔT_C_ was the only term that was retained after model simplification (Table 1). Hence, equal values of ΔT_C_ gives equal probabilities of egg rejection in both species. Moreover, since contrast in volume or shape does not seem to affect rejection probability, this indicates that the host discrimination function (Figure 2) approximates the hosts' responses to real cuckoo eggs. Figure 2 shows the relationship between rejection probability and ΔT_C_ for the pooled data.

**Table 1:** Generalized linear models of the probability of accepting congeneric parasitic eggs in chaffinches and bramblings.

Parameter	Df	Deviance	P
ΔT_C_	1	52.8	< 0.0001
ΔT_B_	1	3.48	0.06
Species	1	0.43	0.51
Shape	1	0.35	0.55
Volume	1	0.33	0.57
ΔT_B_ × Species	1	2.40	0.12
Volume × Species	1	1.51	0.22
ΔT_C_ × Species	1	0.65	0.42
ΔT_B_ × ΔT_C_	1	0.15	0.70
Shape × Species	1	0.03	0.87

Stepwise backward deletion was used to simplify the global model including all parameters, and significance of parameters was evaluated by the change in deviance between models with and without the parameter in question. ΔT_C_ is the only term included in the minimal adequate model. ΔT_B_ denotes brightness contrast, and was calculated according to [25]. Volume and shape was calculated according to [86] and [87], respectively. Rejection rates were 0.60 (N = 151) for the chaffinch and 0.66 (N = 137) for the brambling. Mean (SD) of ΔT_C_ and ΔT_B_, respectively, was 0.044 (0.023) and 6.48 (4.20) for the chaffinch and 0.051 (0.031) and 5.52 (4.01) for the brambling.

### Natural Parasitism and Appearance of Cuckoo Eggs in Relation to Optimum

The distribution of pair wise color contrasts (ΔT_C_) were similar in all populations studied (Table 2), meaning that interclutch variation in egg coloration is of similar magnitude. The average mimicry (ΔT_C_) of the best achievable mimetic eggs was also similar. Accordingly, the average acceptance probabilities of optimal cuckoo egg types are of the same magnitude across populations and species (i.e. around 50%, Table 2).

**Table 2:** Summary statistics describing different aspects of the clutch color distributions of the brambling and chaffinch.

Population/Sample	N	Color contrast (ΔT_C_) max, mean (SD)	Saturation mean (SD)	Average color contrast (ΔT_C_) of optimal cuckoo egg types	Average rejection rate of optimal cuckoo egg types (SD)
Brambling					
Field data (2007–2008)					
Tydal	47	0.136, 0.047 (0.025)	0.109 (0.019)	0.034 (0.017)	0.53 (0.16)
Kittila	88	0.177, 0.044 (0.025)	0.100 (0.018)	0.031 (0.018)	0.50 (0.16)
Ammarnäs	97	0.159, 0.043 (0.025)	0.102 (0.016)	0.031 (0.017)	0.50 (0.16)
Fennoscandia	232	0.180, 0.045 (0.025)	0.103 (0.018)	0.032 (0.018)	0.51 (0.16)
Museum data (1881–1940)					
Fennoscandia	343	0.173, 0.040 (0.024)	0.106 (0.017)	0.028 (0.017)	0.47 (0.16)
Chaffinch					
Field data (2007–2008)					
Stjørdal	157	0.152, 0.045 (0.027)	0.070 (0.016)	0.033 (0.018)	0.52 (0.17)
Museum data (1881–1940)					
Great Britain	273	0.151, 0.044 (0.025)	0.095 (0.022)	0.032 (0.017)	0.51 (0.16)
Sweden & Denmark	273	0.143, 0.041 (0.024)	0.089 (0.015)	0.030 (0.016)	0.49 (0.15)
Finland, Karelen (Russia) & Estonia	79	0.120, 0.042 (0.024)	0.089 (0.017)	0.031 (0.014)	0.50 (0.14)
Great Britain and Fennoscandia	625	0.173, 0.043 (0.025)	0.091 (0.019)	0.031 (0.016)	0.50 (0.15)

Optimal cuckoo egg types equal the host egg type which achieves the lowest ΔT_C_ value/rejection probability when averaged over all possible pair wise combinations in which the egg features. Rejection rates are predicted from a univariate logistic regression of ΔT_C_ on the probability of rejecting a parasitic egg (Figure 2).

**Figure 3:**
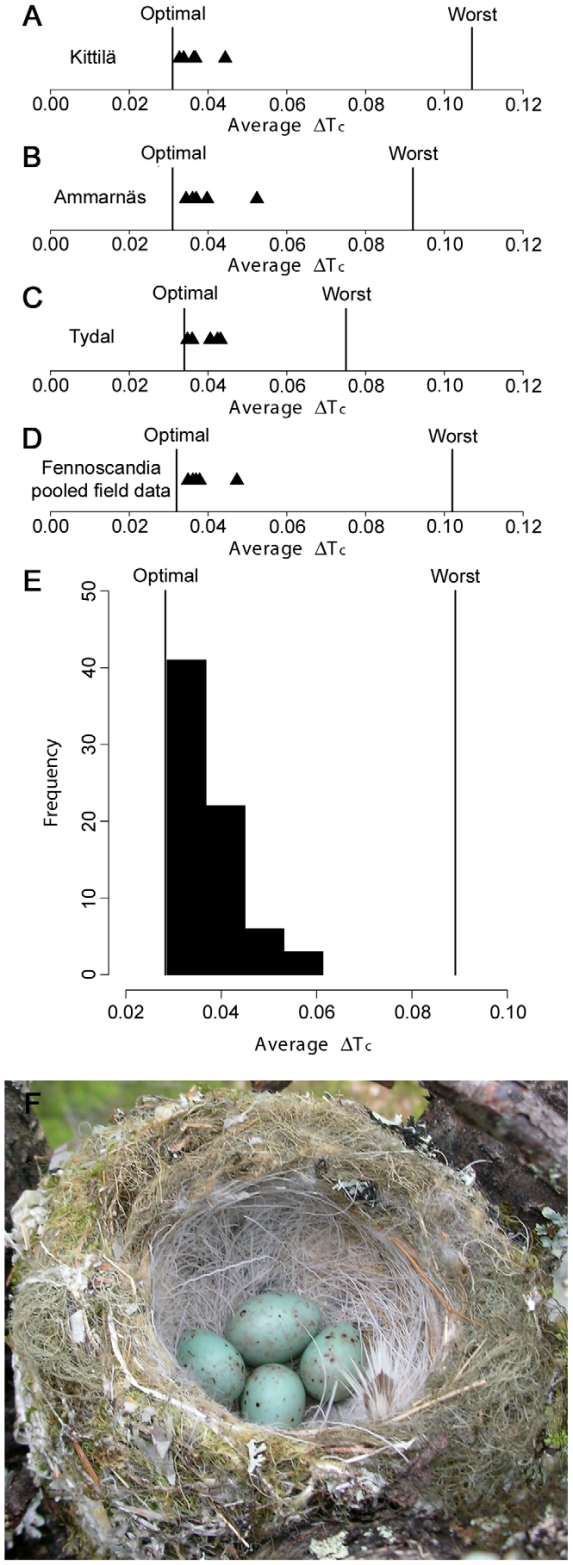
Colors of cuckoo eggs in relation to predicted optimum for parasitism of bramblings. Black triangles (A–D) and black bars (E) indicate the mean color contrast of cuckoo eggs resulting from comparison with all brambling clutches in a population. The leftmost vertical line in each figure indicates the lowest average color contrast that can be achieved, given the color distribution of the host's clutches. The rightmost vertical line in each figure indicates the highest average color contrast a cuckoo egg could have, and still be a perfect match to at least one of the host clutches in the population. Cuckoo eggs in A–D were all found in Kittilä (N = 5), whereas cuckoo eggs in e were measured in museums (N = 72). A: Kittilä (N = 88 host clutches). B: Ammarnäs (N = 97 host clutches). C: Tydal (N = 47 host clutches). D: Kittilä, Ammarnäs and Tydal pooled (N = 232 host clutches). E: museum clutches collected in the period 1841–1979 (N = 343 host clutches). F: a brambling clutch with three host eggs and one cuckoo egg (top).

The cuckoo eggs measured in the field in Kittilä had colors that were very close to the optimum for maximizing acceptance by brambling hosts. The same cuckoo eggs would also be close to the optimum in two remote unparasitized populations (Figure 3A–D). When analyzed at the scale of Fennoscandia, museum *Fringilla-*type cuckoo eggs also tended to have optimal colors for parasitizing bramblings (Figure 3E). Furthermore, there was no correlation between geographical distances and color distances for brambling eggs (Table 3). This corroborates the field data by suggesting that the optimal egg colors for cuckoos do not vary noticeably within Fennoscandia. Accordingly, there was no correlation between geographical distances and color distances for cuckoo eggs (Table 3), indicating that *Fringilla* type cuckoo eggs do not show any marked geographical variation in color distribution within Fennoscandia.

**Table 3:** Mantel and partial Mantel tests.

Dataset	Nr clutches	Mean (SD)		Matrix comparison	r	P
		Year	Geo			
Chaffinch	569	27 (20)	836 (569)	Geo-color (year)	0.061	0.001
				Geo-color	0.062	0.001
Chaffinch, Fennoscandia	339	27 (20)	517 (361)	Geo-color (year)	0.039	0.019
				Geo-color	0.046	0.008
Chaffinch, Sweden and Denmark	320	26 (21)	320 (230)	Geo-color (year)	0.055	0.013
				Geo-color	0.068	0.007
Brambling, Fennoscandia	330	29 (23)	393 (266)	Geo-color (year)	−0.018	0.74
				Geo-color	−0.015	0.75
Cuckoo	67	23 (18)	415 (260)	Geo-color (year)	0.021	0.36
				Geo-color	0.034	0.29

Mantel tests were performed on matrixes of geographical distances (geo) and color space distances (color), of chaffinch, brambling, and cuckoo eggs. The parentheses indicate that year of collection is controlled for in a partial Mantel test.

Among the museum cuckoo eggs found in chaffinch clutches, only 22% (N = 45) could be classified as a *Fringilla* type cuckoo egg. In comparison, 78% (N = 55) cuckoo eggs found in brambling clutches were *Fringilla* type. These numbers are in close agreement with a previous study [22] which classified 12% (N = 76) of cuckoo eggs in chaffinch clutches as *Fringilla* type compared to only 77% (N = 53) of cuckoo eggs in brambling clutches.

### Egg Color Distributions of Brambling and Chaffinch

The distribution of clutches in the color tetrahedron differed significantly between the chaffinch and brambling, considering both field data (Euclidean distance MRPP on xyz-coordinates, P<0.001, K = 0.12) and museum data (Euclidean distance MRPP on xyz coordinates, P<0.001, K = 0.03).

**Figure 4:**
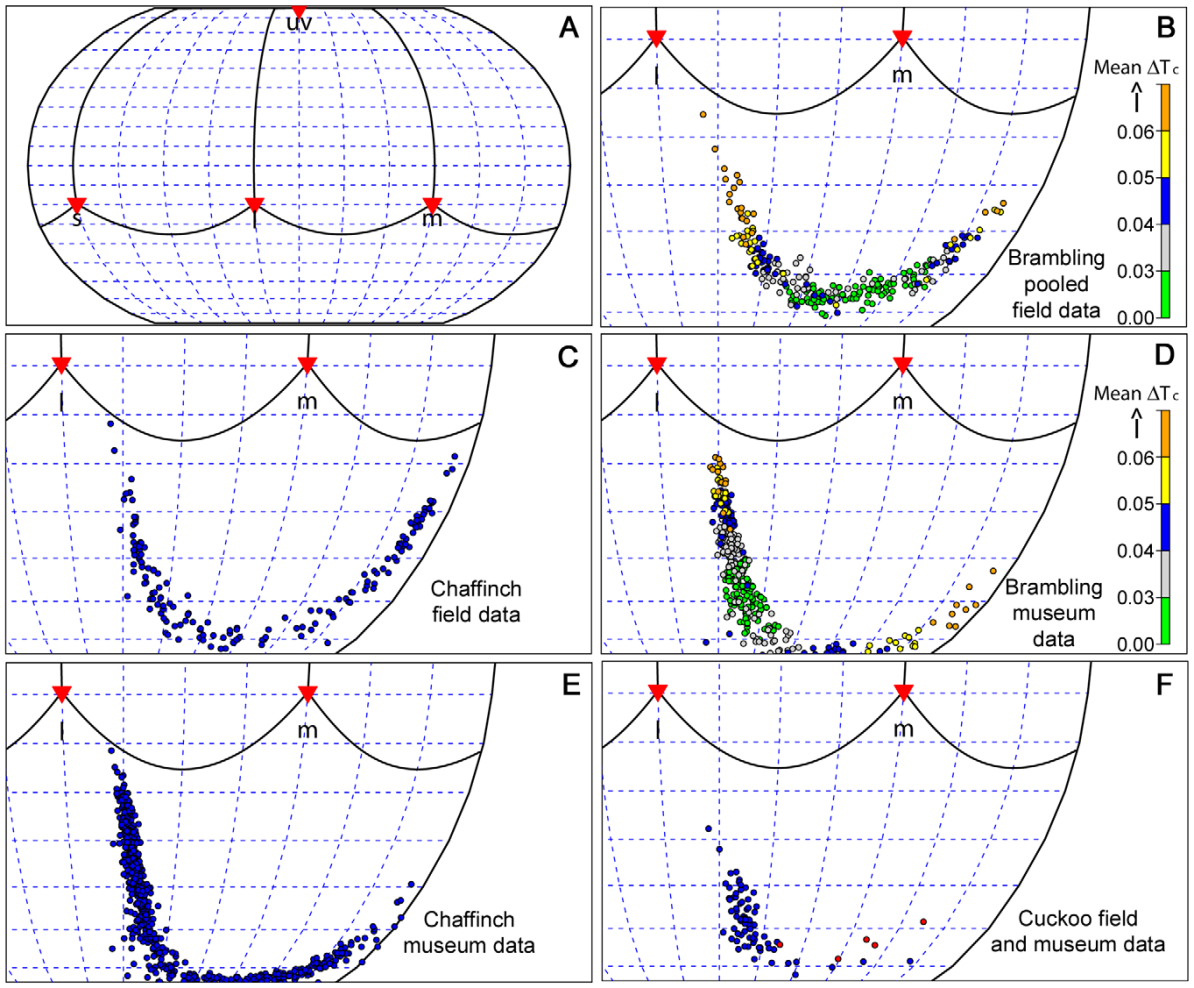
Color (hue) distributions of brambling, chaffinch and cuckoo eggs. (See main text for explanation of Robinson projections). A: the entire projected tetrachromatic hue-space for reference. Red triangles indicate the projections of the ultraviolet (uv), short (s), medium (m) and long wavelength (l) vertices of the tetrahedron. Solid lines indicate the projections of the four edges connecting the different vertices. B–C: the distribution of hues for 232 brambling (pooled clutches from Kittilä, Ammarnäs and Tydal), and 157 chaffinch clutches (Stjørdal), respectively. D–E: the distribution of hues for 343 brambling (Fennoscandia) and 625 chaffinch clutches (Great Britain and Fennoscandia) held in various museum collections. F: the distribution of hues for 72 museum cuckoo eggs classified as *Fringilla* egg morphs (blue color) and five fresh cuckoo eggs found in brambling nests in Kittilä in the period 2005–2008 (red color). For plotting purposes only, three clutches in E located close to the s-vertice (i.e. bluish hues) were slightly transformed in order to make them appear within the plotted frame. Note the overlap between the hue-spaces occupied by the two hosts (B–E). The circles in B and D have different colors to illustrate the direction of selection on hue under the assumption that cuckoo egg color distributions are given by the five and 72 cuckoo eggs in Figure F, respectively. Different colors indicate differences in the average color distance (ΔT_C_) between host and cuckoo eggs. Parasitism clearly imposes disruptive selection, and an eventual evolutionary response is therefore predicted to change the distribution towards stronger bimodality, whereupon it would converge with the chaffinch's hue distribution (C, E).

The Robinson projections of hues illustrate the patterns of variation in hue independent of saturation (Figure 4). The distribution of hues differed significantly between chaffinch and brambling clutches, considering both field data (Euclidean distance MRPP on θ, Φ, P<0.001, K = 0.02, Figure 4B–C) and museum data (Euclidean distance MRPP on θ, Φ, P<0.001, K = 0.01, Figure 4D–E). The projections show that a large proportion of the hues are found in both species, except from the most pure blue hues which were not found among brambling clutches collected in the field (Figure 4B–C). The projections also show that the hue distribution of the chaffinch is clearly thicker at the tails (i.e. more bimodal) compared to the brambling (Figure 4B–E), both for field and museum clutches. Accordingly, the variance of the chaffinch's hue distribution is significantly greater than the variance of the brambling's hue distribution (MHGD, field data: df = 1, 387, F = 78.2, P<0.0001; museum data; df = 1, 966, F = 7.80, P = 0.005). The variance of the brambling's hue distribution was similar in the three populations studied (MHGD, df = 2, 229, F = 0.49, P = 0.61).

A bimodal distribution of hues indicates that there are two different clusters in tetrachromatic color space: i.e. all colors are found close to two separate axes running from the centre of the color space. For both finches, the saturation of the clutch colors were approximately normally distributed (Figure S1). In addition, inspection of residual plots suggested that there are no marked non-linearities in the relationship between saturation and hue (Figure S2). Together, this indicates that the multimodal tendencies in the egg colors are found in the hue dimension, which is captured by the Robinson projections.

Compared to fresh clutches, hue distributions of museum clutches were skewed towards the l (red) vertice, and clutches in the blue and blue-green segment have markedly reduced values for the ultraviolet component (Φ) of hue (Figure 4B–E). Preliminary analyses of eggs collected in the field show that the colors of these eggs have changed in the same direction in one year (JR Vikan, unpublished results). For this reason, we will not discuss the differences between museum and field samples any further in this paper.

### Form of Selection Imposed on Brambling Clutches by Cuckoo Eggs

We calculated the average ΔT_C_ of each brambling clutch in order to evaluate the direction of selection on hue. We assumed that the cuckoo egg distribution was given by the *Fringilla* cuckoo egg types measured in this study (treating field and museum data separately), which are close to their optimum color (Figure 3). The calculations indicate that there should be huge opportunities for disruptive selection on hue (Figure 4B, 4D). For example, whereas bramblings with greenish clutches would have an expected probability of rejecting cuckoo eggs of about 50%, the corresponding probability would be 80% or higher for bramblings laying eggs with extreme hues (i.e. reddish-brown or bluish). An evolutionary response in the brambling would therefore transiently change the hue distribution in the direction of stronger bimodality, which in turn would cause the distribution to converge on the chaffinch distribution (Figure 4C, 4E).

### Geographical Variation in Egg Colors

There was a weak but statistically significant correlation between geographic distance and egg color distance in the chaffinch, as revealed by Mantel and partial Mantel tests (Table 3). The correlation remained significant when we restricted the analysis to clutches from Sweden and Denmark (Table 3). The distribution of geographical distances for these clutches is comparable to that of bramblings in Fennoscandia, for which there was no correlation between color distances and geographical distances. In accordance with the Mantel tests, permutational MANOVA's revealed a significant effect of latitude/longitude on chaffinch egg colors (Analyses of distance: Great Britain and Fennoscandia: Latitude: df = 1, F = 43.1, R^2^ = 0.069, P = 0.0001; Longitude: df = 1, F = 32.6, R^2^ = 0.053, P = 0.0001; Fennoscandia: Latitude: d.f. = 1, F = 16.5, R^2^ = 0.046, P = 0.0001; Longitude: df = 1, F = 8.31, R^2^ = 0.024, P = 0.0022; Sweden and Denmark: Latitude: df = 1, F = 17.1, R^2^ = 0.060, P = 0.0001; Longitude: df = 1, F = 10.8, R^2^ = 0.039, P = 0.0004), but not brambling egg colors (Analyses of distance: Latitude: df = 1, F = 1.70, R^2^ = 0.005, P = 0.18; Longitude: df = 1, F = 0.42, R^2^ = 0.001, P = 0.59), or cuckoo egg colors (Analyses of distance: Latitude: df = 1, F = 1.03, R^2^ = 0.015, P = 0.33; Longitude: df = 1, F = 0.57, R^2^ = 0.009, P = 0.54).

In order to assess in what direction the chaffinch egg color distribution changes with latitude/longitude, we compared the brambling's egg color distribution with the chaffinch's distribution from Great Britain, south-western Fennoscandia, and north-eastern Fennoscandia, respectively. Clutches were grouped into south-western or north-eastern Fennoscandia according to the median latitude. The results are quantitatively similar when Fennoscandian clutches are grouped according to the median longitude (because values for longitude and latitude are strongly correlated, r_s_ = 0.75, P<0.0001, Figure 1C). Ranked by effect size (K), brambling clutch colors were most similar to chaffinch clutches from Great Britain (Euclidean distance MRPP on xyz-coordinates, P<0.0001, K = 0.028), less similar to the south-westernmost chaffinch clutches (Euclidean distance MRPP on xyz-coordinates, P<0.0001, K = 0.037), and least similar to north-easternmost chaffinch clutches in Fennoscandia (Euclidean distance MRPP on xyz-coordinates, P<0.0001, K = 0.064). These results were paralleled by the magnitude of difference in hue distributions: the brambling was most similar to the chaffinch from Great Britain (Euclidean distance MRPP on θ, Φ, P = 0.031, K = 0.004), less similar to the south-westernmost chaffinch (Euclidean distance MRPP on θ, Φ, P = 0.005, K = 0.008), and clearly least similar to the north-westernmost chaffinch (Euclidean distance MRPP on θ, Φ, P = 0.0001, K = 0.053, Figure 4D, Figure S3). Finally, the variance of the hue distribution increased significantly from south-west to north-east: (0.59<0.71<1.05, MHGD with Tukey HSD: P<0.029, Figure S3).

### Selection on Cuckoo Egg Color

**Figure 5:**
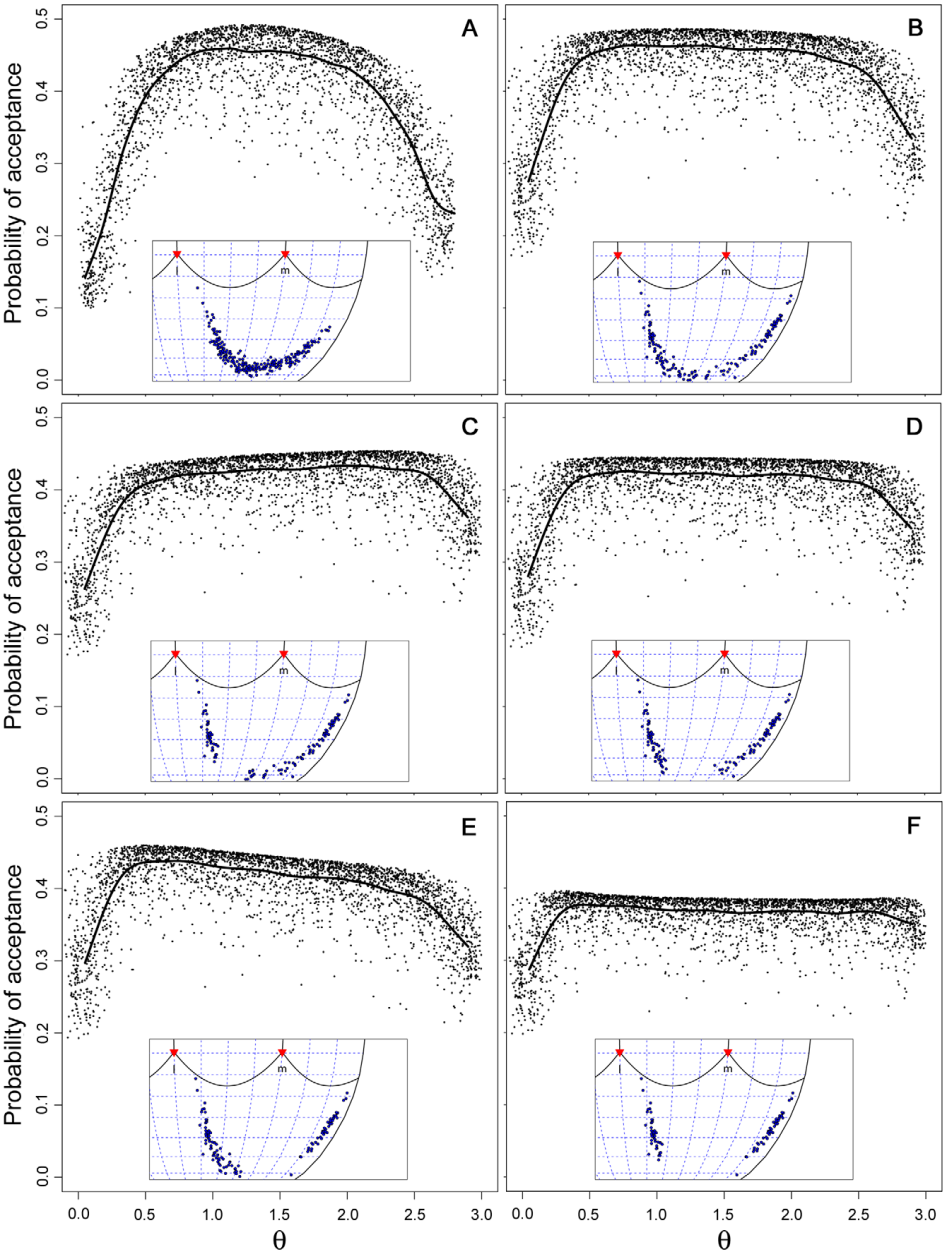
Forms of selection imposed on cuckoo egg colors by different host egg color distributions. Forms of selection on the longitudinal (θ) hue component of hypothetical cuckoo eggs imposed by brambling (A) and chaffinch (B–F) egg distributions. The curves are drawn by cubic spline interpolation and are based on 3000 egg colors that were drawn from within the tetrachromatic color space of each of the hosts to obtain a uniform distribution of θ-values. The mean acceptance probability of each cuckoo egg is based on matching with 232 brambling clutches (A), 157 chaffinch clutches (B), and various subsets of the 157 chaffinch clutches (C–F). Insets show the hue distribution (Robinson projection) of the host clutches in each case. C–F indicates the effect of an increase in the bimodality or skew of the chaffinch's hue distribution. C: chaffinch clutches with θ-values between 30th and 50th percentiles excluded. D: clutches with _0_-values between 40th and 60th percentiles excluded. E: clutches with 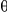-values between 50th and 70th percentiles excluded. F: clutches with θ-values between 30th and 70th percentiles excluded.

To resolve if there are any fine details in the form of selection on cuckoo egg colors, we calculated the mean acceptance probability of a large number of hypothetical cuckoo egg colors randomly selected from within the tetrahedral color spaces occupied by the two hosts, respectively. Figure 5 (A–B) shows the forms of selection imposed on the longitudinal (θ) component of hue by the two host distributions (selection on the latitudinal component is always directional, and selection on saturation is stabilizing towards the same value for all θ). The results show that selection on cuckoo egg colors would be mainly stabilizing, but with a wider plateau in the chaffinch case. To investigate if distinct adaptive peaks could evolve, i.e. giving rise to two distinct cuckoo egg morphs, we defined subsets of the chaffinch distribution that had stronger bimodality and re-calculated the curves. Although increasing bimodality had the expected effect of reducing the average acceptance probability, it did not give rise to distinct peaks (Figure 5C–E).

## Discussion

Although cuckoo-host interactions are famously known as mimicry systems [20], [21], [62], [63], [64], most of the attention to date has been on the evolutionary trajectories of egg recognition, whereas the dynamic aspects of mimicry have been largely neglected in the major discussions [65], [66], [67].

In the present study, we have shown that cuckoos specializing on bramblings have evolved egg colors that are close to maximizing acceptance rate of their eggs, and that this in turn creates ample opportunity for disruptive selection on host egg color. One important question is whether this situation could reflect a matching equilibrium (i.e. equal mean phenotypes). Theoretically, coevolution leads to a matching equilibrium if the response to selection, which is proportional to the additive genetic variance for the trait and the intensity of selection acting on it, is stronger for the parasite than the host [12], [13], [18]. Given the brambling's pervasive lack of site fidelity, the evolutionary unit is likely to be very large, possibly comprising the whole regional population. In this situation, the average parasitism rate across the whole region would determine the selection pressure on brambling egg color (i.e. a panmictic model would be appropriate). Vast numbers of bramblings breed in subalpine birch forests in Fennoscandia, areas for which there exist not even a single report of parasitism [25], [28]. Therefore, although it is possible that parasitism rates can be high locally, the proportion of the entire Fennoscandian brambling population being parasitized is likely to be very small at present. By contrast, cuckoos are subject to strong selection everywhere because bramblings possess similar egg discrimination capabilities throughout Fennoscandia [25]. For this reason, the reciprocal selection pressures acting on egg color are likely to be strongly asymmetric in the brambling-cuckoo interaction. An analogous situation seems to be found in the interaction between the parasitic *Maculinea* butterfly and its ant host *Myrmica ruginodis*, where high levels of gene flow in *M. ruginodis* produces coevolutionary cold spots by cancelling out any selection imposed by the patchily distributed *Maculinea* [4].

There is some indication of consistent regional differences in parasitism of bramblings, with Finnish Lapland being a potential co-evolutionary hot spot (Figure 1C). Although parasitized brambling populations should experience considerable variance in relative fitness related to egg color (Figure 2B, 2D), cuckoo eggs appear to be equally well adapted to each of three distant brambling populations (Figure 3). Moreover, regarding the museum data, we found no correlation between geographic distances and egg color distances within Fennoscandia, either for the brambling or the cuckoo. Our results therefore jointly agree with the predicted effects of lack of site fidelity in the brambling, and are consistent with analyses of geographical variation in other traits [25]. Therefore, as long as parasitism of bramblings is patchily distributed (i.e. the global parasitism rate is low) nomadic behaviour could promote long term persistence of the interaction since the optimum for the cuckoo does not change over time (or at most very slowly) and does not vary between locations in Fennoscandia.

It is also widely acknowledged that gene flow can be a creative force which provides new genetic variation for reciprocal selection, and that the antagonist with the higher amount of gene flow therefore has a general advantage [68], [69], [70]. However, this requires that there are relatively low levels of migration among structured populations, a condition which is not met in the nomadic brambling [42], [43], [44]. Moreover, our analyses suggest that lack of variation is not what limits adaptive evolution in the brambling because parasitized and non-parasitized populations show the same variance in egg color even though there should be a strong opportunity for selection to increase variance in parasitized host-populations. In addition, since the brambling does not show notable geographical variation in egg color, this suggests that random gene flow does not increase the variation available for selection. In coevolving cuckoo-host interactions in general, at least a minimum amount of dispersal might be important for introducing new variation to cuckoo populations because selection is stabilizing on average. On the other hand, dispersal might be less important for the host because selection is on average disruptive in form and therefore normally acts to maintain or increase variation [17].

The current hue distribution of the chaffinch seems to be a good prediction for how an evolutionary response to parasitism would initially affect the brambling's hue distribution. Interestingly, the bimodal tendency in the chaffinch's distribution does not create distinct adaptive peaks for cuckoo egg color, even if the tendency is strengthened. This result is a direct consequence of the egg discrimination abilities being too weak and/or the color distance between the most common host clutches being too small. Therefore, for the present level of host egg discrimination and host egg color variance, evolution of distinct cuckoo egg color polymorphism is an unlikely outcome in *Fringilla*-cuckoo systems. Instead, polymorphism in the cuckoo would be realized in the sense that a broad continuous segment of cuckoo egg colors simultaneously enjoy similar fitness (Figure 5). Therefore, if the brambling experienced a marked increase in regional parasitism rate, we would expect to observe evolution of stronger bimodality in the brambling accompanied by an increased range of cuckoo egg types distributed in between. Accordingly, we can predict that cuckoos specializing exclusively on chaffinches will show a broader range of hues than cuckoos specializing exclusively on bramblings (provided these chaffinches have the same egg discrimination skills and range of clutch colors as Fennoscandian chaffinches). Importantly, although a large fraction of the cuckoo eggs might not resemble any host clutch in this situation, this would not reflect a true evolutionary lag on part of the cuckoo. Marked and parallel polymorphisms have purportedly evolved in some African and Asian cuckoos and their hosts [20], [48], [71], and it would be valuable to examine in more detail what ecological, behavioural and genetic conditions underlie these contrasting outcomes.

While there was no notable geographical variation in brambling egg colors, there was a clear but weak longitudinal/latitudinal cline in the egg color distribution of the chaffinch: the chaffinch becomes increasingly dissimilar to the brambling towards the north-east. This could reflect a historical geographical gradient in parasitism pressure. For example, it is possible that parasitism of chaffinches has historically been more prevalent towards the north-east, where most of the *Fringilla* cuckoo eggs hitherto have been found, and where the breeding ranges of chaffinch and brambling overlap (Figure 1C). Given that chaffinches exert intense discrimination against non-mimetic eggs, it is unlikely that cuckoos adapted to other hosts than the brambling are capable of colonizing chaffinch populations successfully. The geographical differences could alternatively reflect a gradient in some other unidentified climate related selection pressure (e.g. [72], [73]). Regardless of the cause, this particular result shows that degree of site fidelity is likely to influence the extent to which egg colors evolve in response to any selection pressure. Bramblings might also impose stronger limitation on cuckoo recruitment because of a shorter breeding season and larger fluctuations in local abundance and breeding success [29], [30], [74], [75]. Therefore, all else being equal, chaffinches might overall have been subject to higher historical parasitism pressures, which could also have contributed to the observed differences in egg color distribution. The more bimodal egg color distribution of the chaffinch could also potentially be explained by stabilizing selection pressures acting more strongly on brambling egg color than on chaffinch egg color. Intermediate (green) egg colors could for example provide better crypsis than extreme egg colors (reddish or bluish), and the benefits of such crypsis could be higher for bramblings than for chaffinches. However, there is limited experimental support for the possibility that predators of arboreal nests cue on egg color [11], and available data does not suggest that nest predation rates are consistently different in the two species [27]. Detailed studies of the reproductive success of individuals with different egg colors would be valuable.

In summary, our study portrays two closely related cuckoo host species that have evolved different clutch color characteristics, most likely because differences in ecological features have promoted different coevolutionary trajectories. In particular, it is likely that the nomadic behavior of the brambling contributes to tilt the outcome in favor of the cuckoo in terms of a matching equilibrium. In contrast, the marked site fidelity of the chaffinch might have facilitated a stronger counter-adaptation to adapting cuckoos, resulting in a more bimodal egg color distribution than found in the brambling. In this connection, it is relevant to note that reports of regularly parasitized chaffinch populations are strikingly absent [20], [22], [76], [77], [78]. One contributing explanation could be that cuckoos specializing on chaffinches often experience episodes of reduced fitness due to rapid evolution in the host's egg colors. This could occur either because of an increase in host variance (Figure 5) and/or because directional evolution in the host temporally displaces local cuckoo populations from their adaptive peaks [17], [79], [80], [81]. In comparison, we could predict that the brambling-cuckoo interaction is relatively more stable because the adaptive landscape of the cuckoo is likely to change less and more slowly in this interaction. Unfortunately, as for most cuckoo-host interactions there is at present no data to test predictions about epidemiology. Moreover, it is an open question whether cuckoo parasitism could drive directional evolution in *Fringilla* egg colors. This important outcome has not yet been demonstrated in any brood-parasite host system. As regards the present study, the relatively weak geographical differences found in the chaffinch's egg color distribution could be taken to suggest that directional evolution has been of minor importance during past episodes of parasitism. Broad geographical comparisons that control for historical population relationships represent a complementary approach to multigenerational studies since they allow us to sample across a range of histories and conditions [82]. Such approaches have made important contributions to our understanding of evolution of egg recognition [83], [84], and should now be increasingly applied to ongoing interactions in order to clarify whether coevolution drives population differentiation in egg phenotypes and whether cuckoos are normally locally adapted to their hosts in geographically structured interactions [85].

## Supporting Information

Figure S1 **Saturation of cuckoo and** ***Fringilla*** **eggs.** Distribution of saturation of brambling (A, B), chaffinch (C, D) and cuckoo (E, F) eggs. Left column (A, C, E) gives the distribution of saturation for fresh clutches. Right column (B, D, F) gives the distribution of saturation for clutches from museum collections.
(TIF)

Figure S2 **Plots of residuals versus predicted values from linear regressions of saturation on hue (longitudinal component).** A: Brambling clutches collected in the field; B: chaffinch clutches collected in the field; C: Brambling clutches from museum collections; D: Chaffinch clutches from museum collections. The residual plots indicate that there are no abrupt non-linearities or discontinuities in the relationship between saturation and hue.
(TIF)

Figure S3 **Geographic variation in hue of chaffinch clutches.** A: the distribution of hues for chaffinch clutches from Great Britain. B-C: the distribution of hues for chaffinch clutches south and north of the median latitude in Fennoscandia, respectively (see main text and Figure 4 for explanations). For plotting purposes only, three clutches in C located close to the s-vertice (i.e. bluish hues) were slightly transformed in order to make them appear within the plotted frame.
(TIF)
