# Supplementary figures and images for: Outcomes of Brood Parasite–Host Interactions Mediated by Egg Matching: Common Cuckoos Cuculus canorus versus Fringilla Finches
Source: PLoS One. 2011 Apr 29;6(4):e19288. doi: 10.1371/journal.pone.0019288 (PMC3084821; doi:10.1371/journal.pone.0019288)

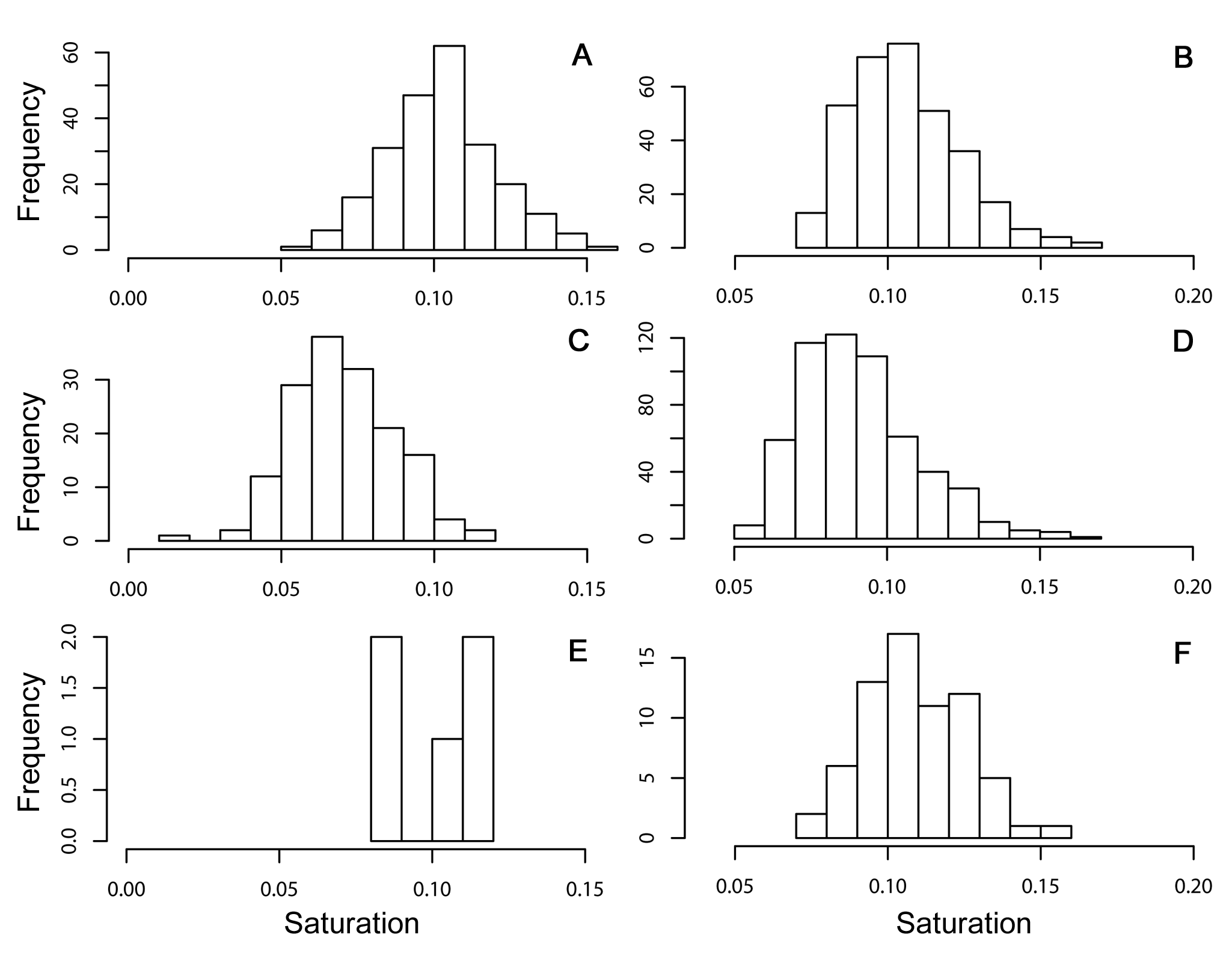

Supplement: Figure S1 — Saturation of cuckoo and Fringilla eggs. Distribution of saturation of brambling (A, B), chaffinch (C, D) and cuckoo (E, F) eggs. Left column (A, C, E) gives the distribution of saturation for fresh clutches. Right column (B, D, F) gives the distribution of saturation for clutches from museum collections. (TIF) [file pone.0019288.s001.tif]

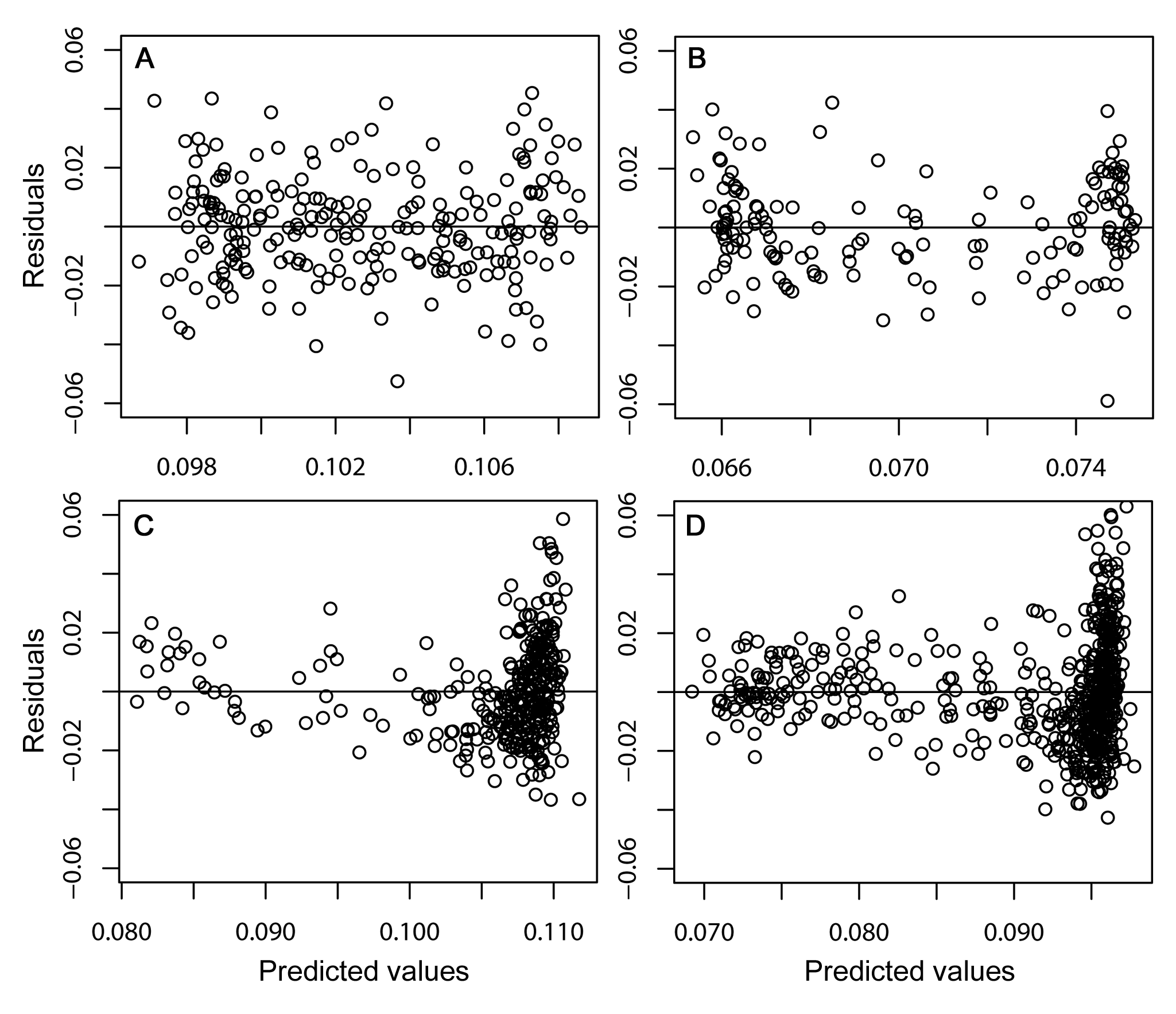

Supplement: Figure S2 — Plots of residuals versus predicted values from linear regressions of saturation on hue (longitudinal component). A: Brambling clutches collected in the field; B: chaffinch clutches collected in the field; C: Brambling clutches from museum collections; D: Chaffinch clutches from museum collections. The residual plots indicate that there are no abrupt non-linearities or discontinuities in the relationship between saturation and hue. (TIF) [file pone.0019288.s002.tif]

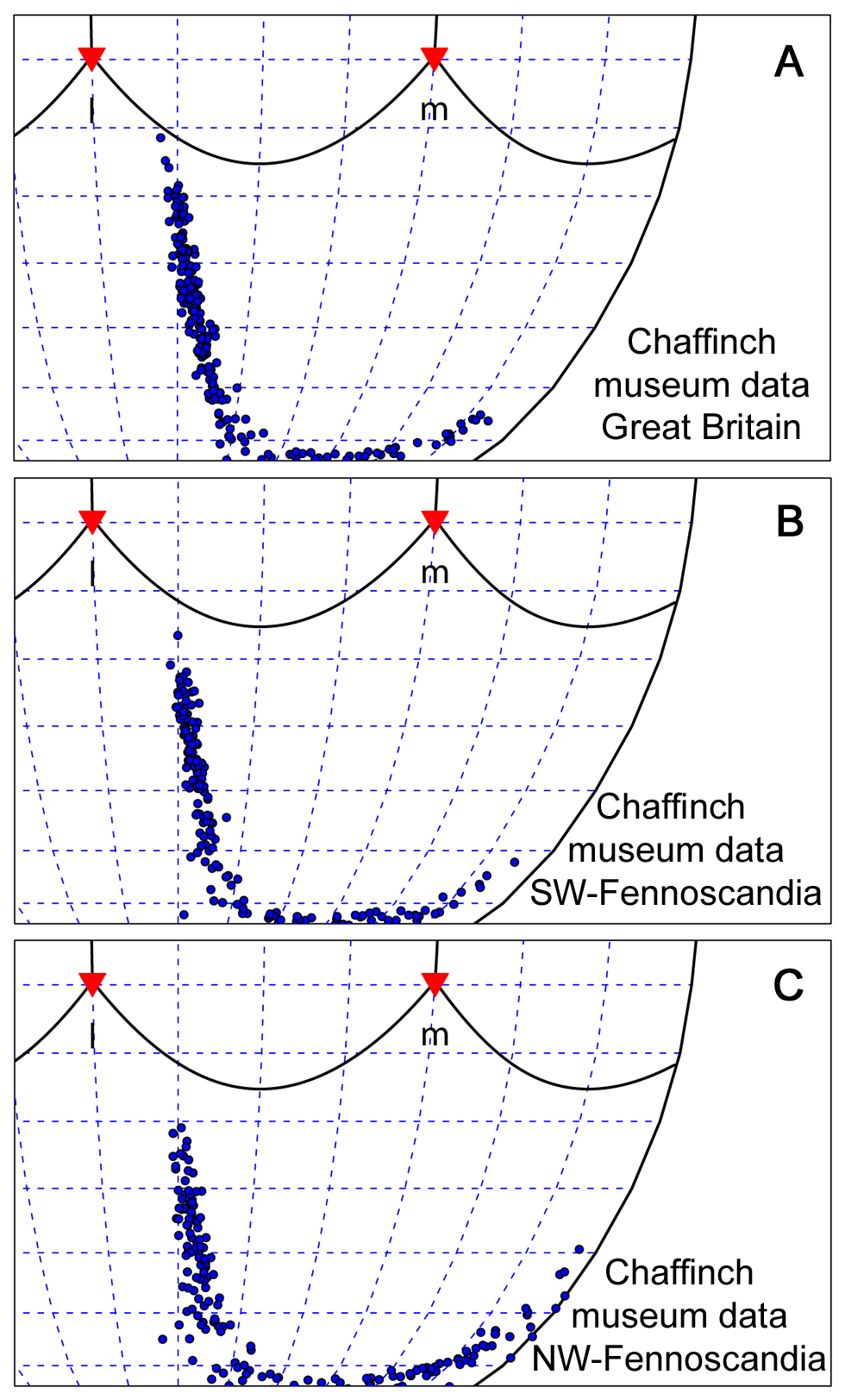

Supplement: Figure S3 — Geographic variation in hue of chaffinch clutches. A: the distribution of hues for chaffinch clutches from Great Britain. B-C: the distribution of hues for chaffinch clutches south and north of the median latitude in Fennoscandia, respectively (see main text and Figure 4 for explanations). For plotting purposes only, three clutches in C located close to the s-vertice (i.e. bluish hues) were slightly transformed in order to make them appear within the plotted frame. (TIF) [file pone.0019288.s003.tif]
